# Laparoscopic uterine graft procurement and surgical autotransplantation in ovine model

**DOI:** 10.1038/s41598-019-44528-1

**Published:** 2019-05-30

**Authors:** Francisco Miguel Sánchez-Margallo, Belén Moreno-Naranjo, María del Mar Pérez-López, Elena Abellán, José Antonio Domínguez-Arroyo, José Mijares, Ignacio Santiago Álvarez

**Affiliations:** 1 Laparoscopy Department, Jesús Usón Minimally Invasive Surgery Centre, 10071 Cáceres, Spain; 2 Microsurgery Department, Jesús Usón Minimally Invasive Surgery Centre, 10071 Cáceres, Spain; 3 Instituto Extremeño de Reproducción Asistida (IERA), 06006 Badajoz, Spain; 4 Assisted Reproduction Unit, Jesús Usón Minimally Invasive Surgery Centre, 10071 Cáceres, Spain; 5 Anatomy and Cell Biology Department, School of Medicine, University of Extremadura, 06071 Badajoz, Spain

**Keywords:** Infertility, Preclinical research, Translational research

## Abstract

Currently, uterus transplantation (UTx) is a clinical option for infertile women. Over the past three decades, treating benign or malignant gynecological diseases with minimally invasive gynecological surgery has improved, providing significant advantages over conventional open surgery. This study addresses the method used for laparoscopic live-donor ovariohysterectomy and graft harvest from a sheep model. Using a microsurgical practice, ten grafts were autotransplanted after uterine perfusion. End-to-end anastomosis techniques were used to approximate veins and arteries. Follow-ups were carried out 2-months after surgery and postoperative studies included ultrasound scan, diagnostic hysteroscopy, vascular angiography, and exploratory laparoscopy. All transplants were completed without complications. After vascular anastomosis, total reperfusion of the tissue was accomplished in all animals without confirmation of arterial or venous thrombosis. Angiographic explorations did not show any statistically significant dissimilarity in the arterial diameters between the different examination times. 3-months after uterine transplantation all animals underwent assisted reproduction techniques. Patent uterine arteries were observed 4, 8 and 12 months after the transplant. 6-months after transplantation, six sheep (60%) became pregnant with assisted reproduction practices. We noticed an increase in the degree of fibrosis of the cervix samples in non-pregnant animals of the transplant group. Laparoscopic surgery can be an advantageous approach for the uterus retrieval procedure during uterine transplantation. However, larger sample sized reports are needed in order to accomplish validation, standardization and wider use of this route.

## Introduction

Although reproductive medicine has improved a lot during the past few decades, there was no treatment available for women with a dysfunctional or absent uterus. Absolute uterine factor infertility (AUFI) in women had no other options for being mothers but adoption or surrogacy. However, this last procedure is currently banned in many countries^1^.

Uterine transplantation, although is still at an early stage, has gained the potential to become the first real therapeutic option for AUFI. Since 2014, eleven children have been born after uterus transplantation^2–4^. These births were the successful result of a clinical trial of uterus transplantation with uteri from live donors, performed in Sweden by Dr. Brännstroms’ team^5^.

Procuring a uterus from a live donor, entails both the advantages of contributing to a successful procedure, but also disadvantages for the donor, as during the retrieval procedure, the living donor is exposed to the risk of surgical complications. The long duration of surgery implies anesthesiology risks and higher time-related complications^6^. It has been informed that the most time-consuming part of the procedure is the difficult surgical isolation of uterine vessels, particularly the veins^1^.

Further developments of uterus transplantation techniques are likely to decrease the possibilities of surgical complications, and new minimally invasive techniques such as laparoscopic or robotic-assisted surgery can help minimize complications in donor graft procurement^1,7^.

Laparoscopic procedures have progressively been considered as the standard for malignant and benign disease surgical treatment, resulting in a permanent evolution of operative techniques. In terms of outcomes, laparoscopy-assisted surgery compared to open surgery has several advantages. One is video magnification, which provides surgeons with better exposure of the organs and their proximate vessels. This allows more accurate and gentle movements to protect these structures during dissection and surgery^8^. In addition, overall skin and muscle trauma are diminished, which means fewer postoperative pain, shorter hospital stays, and a faster recovery period^9^.

In organ transplantation surgery the incision size needs to be enlarged in order to do the retrieval of the organ but not as much as in open surgery, not losing at all the advantages mentioned before^8^. In other cases, laparoscopic and robotic transvaginal approaches have been described as a possible alternative to conventional laparoscopic nephrectomy or uterus retrieval in living donor patients^10,11^. Another advantage of minimally invasive procedures is that the infection rate is reduced because organic tissues are not exposed to the room air for long periods of time when compared to traditional surgery.

While uterus transplantation has been proved in experimental conditions and performed in twenty-six human patients^5,7,12–15^, further developments are needed before additional human cases can be conducted. For example, to apply a laparoscopic approach to the procurement of graft in living donors, adequate experience in laparoscopic surgery as well as favorable anatomy is required.

Different phases require different models in the development of new surgical techniques. In organ transplantation, non-rejecting models such as autologous or syngeneic models allow surgical techniques to be evaluated without the obstacle of immunosuppression^16^. This was the reason why we chose an autologous model, in which the uterus is removed and retransplanted into the same animal.

The purposes of this study were to evaluate, using laparoscopic and microsurgical techniques, the feasibility and safety of an ovine model for uterine autotransplantation.

## Methods

### Animals and experimental design

The Animal Welfare Ethics Committee of the Minimally Invasive Surgery Centre, endorsed all the preliminary protocols, which totally adhered the recommendations defined by the local government (Junta de Extremadura) and the EU Directive (2010/63/EU), concerning the utilization of laboratory animals for scientific purposes as well as Spanish laws (RD 53/2013), ARRIVE rules and the Guide for Care and Use^17^.

A total of 15 fertile female domestic merine sheep (*Ovis orientalis aries*) weighing 40–50 kg were used for this study. All ewes aged 2–3 years old and were pregnant at least once in their life. Sheep were housed in groups of five, and they were given water *ad libitum* and a standard eating regimen once every day. A basic physical and hematological examination (complete blood count, biochemical analysis) was carried out preoperatively, reporting the animals to be in sound condition before experiencing medical procedure. It was forced overnight fasting for 24 h, yet free admission of water was permitted up to 4–6 h before the procedure.

The animals were arbitrarily splitted into two groups; a control group (CG) of five non-transplanted ewes and an autotransplantation group (AG) of ten individuals.

### Anesthesia and monitoring

The animals received intravenous (IV) induction with propofol 6 mg/kg (Propofol Sandoz 10 mg/ml; Sandoz Farmacéutica S.A., Madrid, Spain) after preoxygenation during 5 minutes, the day the surgeries took place. A cuffed endotracheal tube I.D. no. 9 mm was used to intubate the trachea, and the lungs were kept up in normocapnia by mechanical ventilation by a closed circle anesthetic breathing system (Maquet Flow-i, Maquet Critical Care AB, Solna, Sweden), that lead to a 14 breaths/minute respiratory rate. For anesthetic maintenance, all animals received sevoflurane (Sevorane; Abbott Laboratories, Madrid, Spain) at 1.4 minimum alveolar concentration (MAC) (EtSEV = 3.7%) in oxygen (fresh gas flow 1 litre/minute, FiO2 = 0.5).

Ketorolac 1 mg/kg (Ketorolaco trometamol Normon 30 mg/ml, Madrid, Spain) and buprenorphine 0.01 mg/kg (Bupaq Multidose 0.3 mg/ml; Richter Pharma AG, Wels, Austria) were used for surgical analgesia and for fluid therapy, intravenous saline was maintained at 2 ml/kg/h. Respiratory and heart rates, FiO2, EtCO2, pulseoximetry, tidal volume, inhaled and exhaled anesthesia and airway peak pressure were monitored along the entire procedure.

### Baseline angiography

The animals underwent a selective basal angiography of the uterine arteries the same day of surgery. Under general anesthesia, the ewes were secured in recumbent position on the angiography table, limbs extended cranially and caudally. The abdomen and crotch region were prepared and covered in a sterile manner. The femoral arterial access on either side was established using a modified Seldinger method. The femoral artery (right or left) was perforated with a puncture needle (Entry Needle 18 g × 2–3/4, AngioDynamics, New York, United States) further to the incision made with a scalpel blade (No. 11) at approximately 1 cm caudal to the inguinal ligament. Immediately after the arterial blood return from the needle was seen, a guide wire of a 5Fr introducer sheath set (Check-flo; William Cook Europe, Bjaeverskov, Denmark) was inserted into the femoral artery. The needle was removed whereas the guide wire was maintained in the femoral artery. The 5Fr sheath with dilator was placed in the artery over the guide wire, and then the dilator and guide wire were pulled out, leaving the sheath in place.

With fluoroscopic guidance (BV Pulsera, Philips Healthcare, Best, Netherlands), a 5Fr selective catheter (Sos Omni-I, AngioDynamics, New York, United States) together with a 0.035″ guide wire (Glidewire®, Terumo Medical, Tokio, Japon) was inserted through the percutaneous vascular access to the contralateral external iliac artery. After the guide wire removal, the angiographic catheter was turned and pushed into the abdominal aorta, where the distal end of the catheter was restored to its original configuration. A global angiography was done in the pelvis by manual infusion of 10 ml of contrast agent (76%, Urografin®, Bayer Pharma AG, Berlin, Germany) in the standard back-front position, so as to demonstrate the vascular system of the respective bilateral external and internal iliac arteries and their branches. The angiographic catheter was specifically placed into the internal iliac arteries on both sides, respectively, with the tip of the catheter immediately cranial to the segment where the uterine artery originates. Selective angiography was performed with 5 ml manual injection of the contrast medium, by using the ipsilateral anterior oblique projection of 25°–30°. The catheter and sheath were removed after angiography and hemostasis was obtained by hand compression at the puncture site for 10 minutes. Immediately after, the animals were ready to undergo the transplantation surgery.

### Surgical technique

#### Uterine graft laparoscopic procurement

Carbon dioxide (pneumoperitoneum) was insufflated in the abdomen by a Veress needle. An automatic insufflator maintained intraabdominal pressure at 12 mmHg (Electronic Endoflator, Karl Storz GmbH, Tüttlingen, Germany). At the left and right hemiabdomen, approximately 9 cm from the camera port, a 10-mm and a 5-mm trocar were placed bilaterally under direct visualization. There was another 5-mm trocar in the left iliac fossae before the animals were placed in the Trendelenburg position.

**Figure 1:**
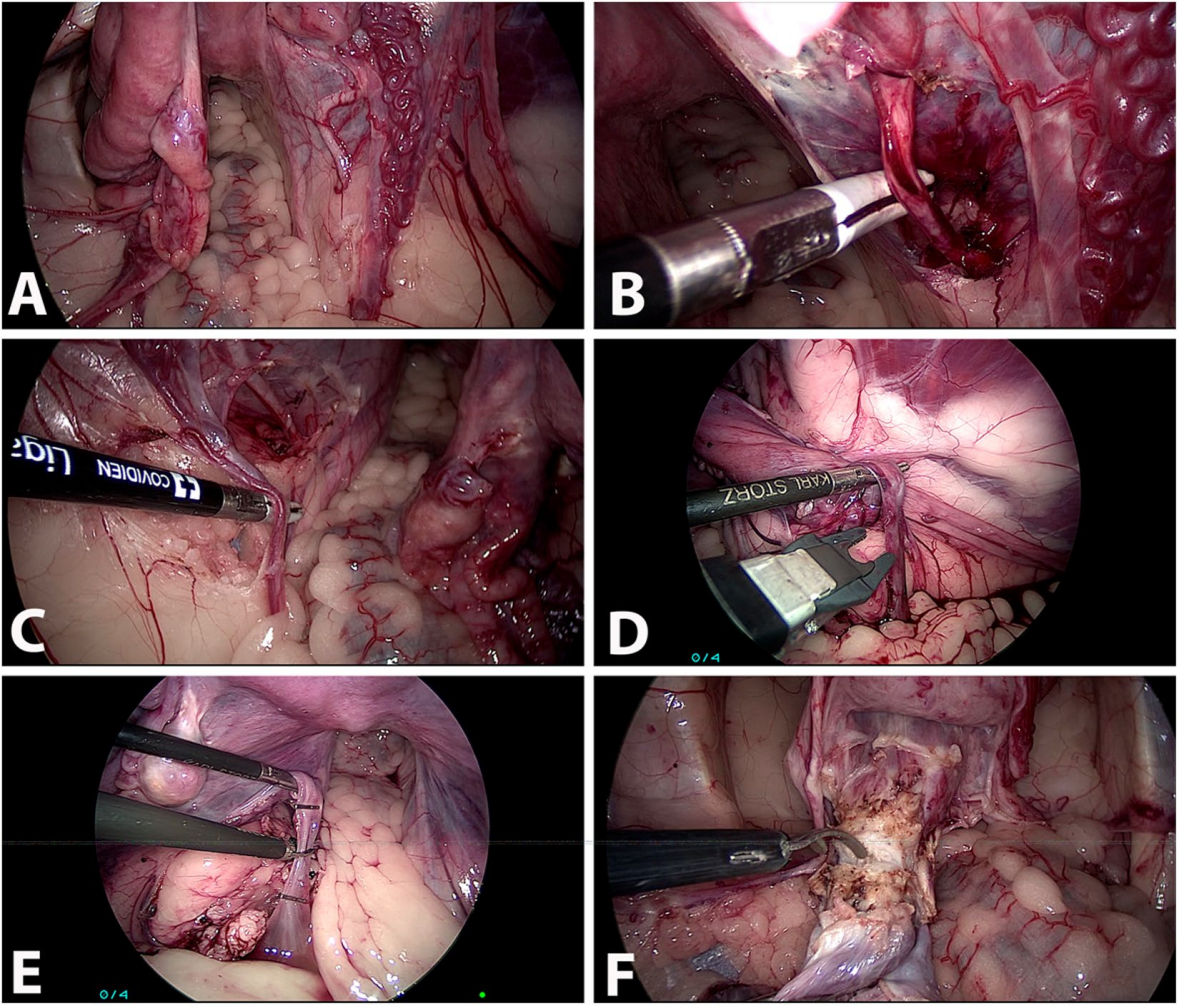
Surgical steps during laparoscopic ovariohysterectomy. (**A**) Laparoscopic exposition of the ovarian and uterine vessels. (**B**) Laparoscopic isolation of the uterine artery. (**C**) Laparoscopic dissection of the utero-ovarian vein. (**D**) Laparoscopic clipping of the utero-ovarian vein. (**E**) Utero-ovarian vein being divided between clips. (**F**) Anterior colpotomy for detaching the uterus from vagina.

After an abdominal and reproductive tract general examination, a laparoscopic total hysterectomy with a bilateral oophorosalpingectomy was carried out. First, the uterine arteries and the utero-ovarian veins were dissected gently and exposed, using maneuvers of blunt dissection and electrocautery (Fig. 1). Secondly, all three suspensory, broad, and round ligaments were transected with a sealing device (Ligasure^TM^, Medtronic, Minnesota, United States). Then, the vessels were clamped with titanium clips and were cut with a laparoscopic scissor. However, five minutes prior to the vessels’ occlusion, heparin was intravenously administered every hour (100UI in the first dose, and a half of the amount in the following) until the autotransplant was completed. Finally, the cervix was transected using the laparoscopic sealing device.

When the whole specimen was isolated, the optic trocar’s incision was elongated in order to remove the graft.

### Graft preparation

**Figure 2:**
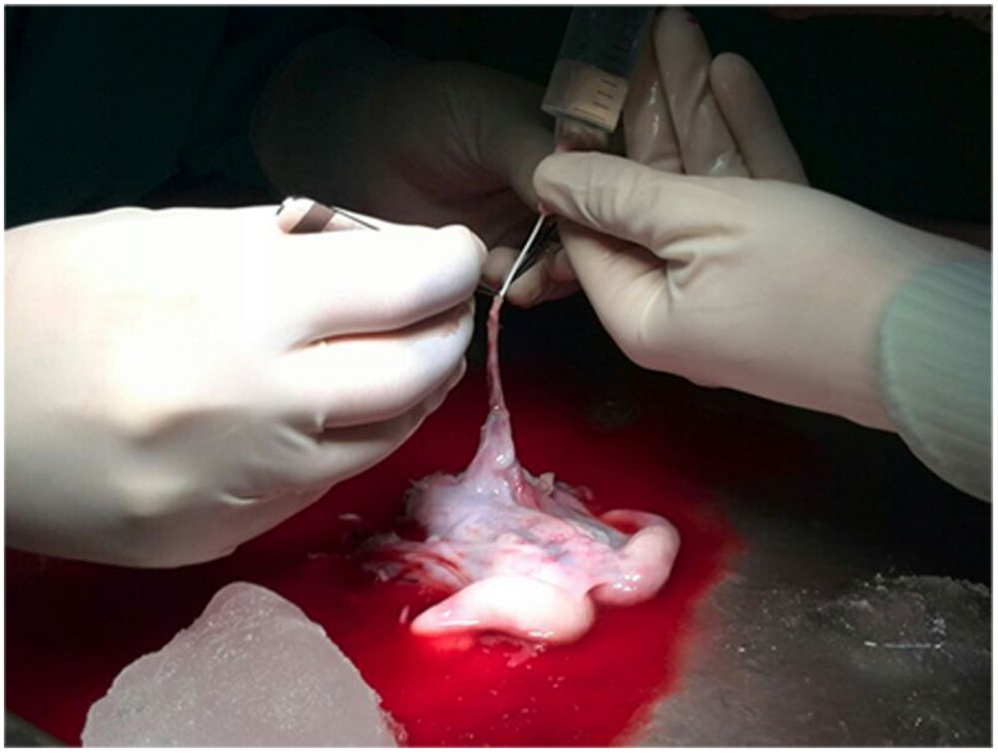
Graft preparation: manual flushing with chilled heparinized saline solution.

The uterus was brought immediately to a back-table, it was stored in a sterile tray and chilled with ice at 4 °C. The organ was manually flushed with chilled heparinized saline solution through each uterine artery, using a 20 G I.V. catheter until the organ was blanched and clear fluid drained from the utero-ovarian veins (Fig. 2).

### Autotransplantation surgery

**Figure 3:**
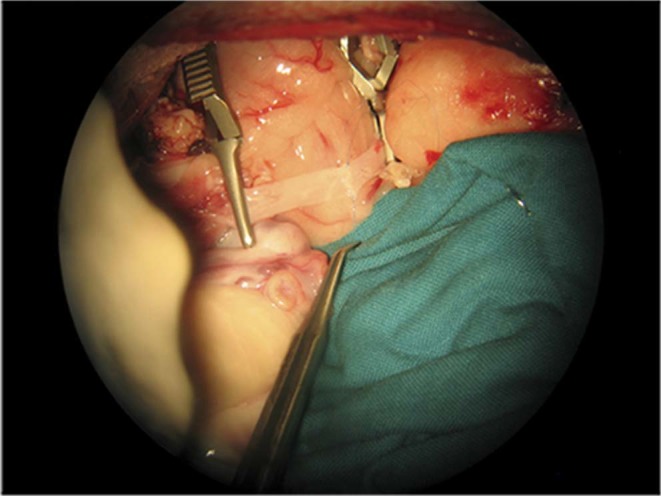
End-to-end anastomosis of a uterine vein.

**Figure 4:**
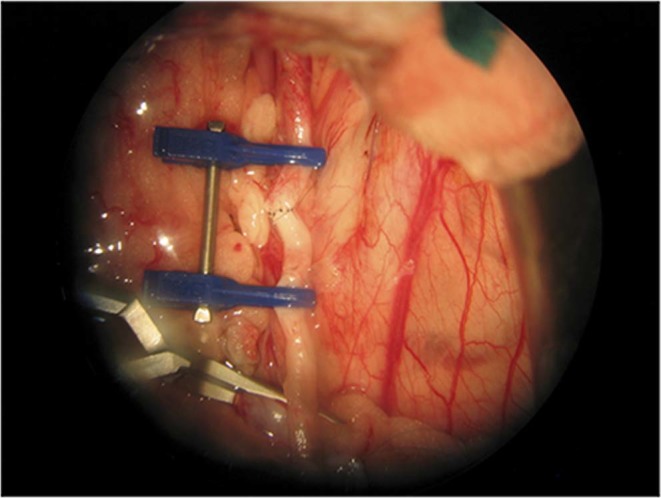
End-to-end anastomosis of a uterine artery.

To avoid a long cold ischemia period, preparatory surgery of the edges of the vessels in the patient was initiated. A surgical microscopy (OPMI® PENTERO® 800 de ZEISS, Oberkochen, Germany) and 8/0 non-absorbable monofilament sutures (Dafilon®, Braun, Aschaffenburg, Germany) were required for vascular anastomosis. An end-to-end bilateral anastomosis technique with two continuous hemisutures was used to align the veins (Fig. 3), while an end-to-end bilateral anastomosis was used non-continuously to approximate the arteries (Fig. 4). Once the vascular anastomosis was completed, patency was assessed by the strip test (empty and refill test) and the color shift of the uterus, from white to reddish. The cervix was approximated to the vagina with 2/0 (Novosyn®, Braun, Aschaffenburg, Germany) suture in a non-continuous pattern and the uterine ligaments were fixed by simple stiches to the pelvic cavity in order to avoid rotation. Finally, the incision sites for the abdominal wall and trocar were closed.

Intraoperative blood loss, complications and operative times (total dissection), laparoscopic total procurement, time of cold ischemia, of the first period of warm ischemia (I), of the second period of warm ischemia (II), total warm ischemia (I + II), and the total surgery time) were also recorded.

### Post-operative care and follow-up

The animals were fed the night after surgery with hay and the following days with a regular diet. All animals received analgesics as postoperative treatment: buprenorphine (0.02 mg/kg/8 h, during 2 days) and meloxicam (0.2 mg/kg/12 h, during 5 days); antibiotics: enrofloxacin (5–7,5 mg/kg/24 h, during 7 days); a stomach protector: sucralfate (1 g per day, every day) and an anticoagulant: acetylsalicylic acid (1 g per day, every day). Daily physical exams were performed during this follow - up period to detect symptoms of pain or infection. Biochemical and hematological analyses, angiography (as described previously), transvaginal ultrasound, abdominal color Doppler ultrasound, and vaginoscopy were carried out at predetermined time points (15 days, 1 month and 2 months after surgery) in order to assess the general health condition of the animal, the vascular patency and viability of the graft. In addition, an exploratory laparoscopy was also performed one month after surgery.

### Artificial insemination and pregnancy determination

All animals were included in this phase of the study, namely, the autotransplant group animals and the control group animals.

Three months after the surgery, the ewes were stimulated during 14 days using progesterone intravaginal sponges (Sincropart®, Ceva Salud Animal, Barcelona, Spain). The day of sponge removal, each animal was administered with pregnant mare´s serum gonadotropin (PMSG) (Foligon®, MSD, New Jersey, United States) to trigger ovulation. Cervical insemination with fresh semen was accomplished 55 hours after PMSG injection. The presence of pregnancy was confirmed via abdominal ultrasonography on day 40 after the artificial insemination. If the sheep was not pregnant, estrus induction and insemination were repeated once more, with a maximum of three attempts.

### Pregnancy follow-up and Caesarean section delivery

Daily physical exams were performed to detect any abnormalities. Fetal biparietal diameter (BPD) and renal length (RL) were measured by abdominal ultrasound at day 40, 65, 90, 115 and 140 after insemination, to monitor fetal growth and verify that it was in accordance with its gestational age. In addition, fetal heartbeat was also assessed to determine fetal viability.

On estimated pregnancy day 142, under general anesthesia, both transplanted and controlled animals underwent a planned caesarean section (as described before). Vital signs were checked in the newborns, weighed and handled using standard practices of neonatal care.

### Post-mortem analyses and histology

At the end of the study (2 months after delivery), all animals except for the lambs, were euthanized. Postmortem analyses were done immediately after, to look for evidence of abdominal adhesion or any lesion or abnormal tissue in the uterus. For their histological study, the uterine samples were sent to an anatomical pathology laboratory and stained with light green hematoxyline, eosin and Masson’s trichrome.

### Statistical analysis

Values are shown as single values and means ± standard deviation. Statistical analysis was conducted using SPSS 15.0 for Windows (SPSS Inc., Chicago, Illinois) statistics software. To determine the normal distribution of the samples, a Kolmogorov-Smirnov test was performed. With this condition verified, a Student’s t-test was applied with a statistical significance of p ≤ 0.05 to perform a comparison between groups.

## Results

### Outcome of surgery

In all ten cases, laparoscopic procurement surgery was performed without complications. The average transplant procurement duration was 112 ± 22.79 min. The median duration of the whole operation incision to closure was 373.44 ± 47.65 min. The median cold ischaemic period was 59.11 ± 10.62 min. The median of the first phase of warm ischemia and the second phase of warm ischemia were 16.78 ± 2.05 min and 105.56 ± 19.29 min, respectively. The median time of the phase of total warm ischemia was 122.22 ± 20.22 min (Table 1).

**Table 1:** Median duration of the main different phases of surgery during uterus autotrasplantation in the ewe.

Surgical Time	Minutes ± SD
Graft procurement	112 ± 22.79
Total surgery (retrieval + transplant)	373.44 ± 47.65
Cold ischemia	59.11 ± 10.62
First warm ischemia	16.78 ± 2.05
Second warm ischemia	105.56 ± 19.29
Total warm ischemia	122.22 ± 20.22

**Figure 5:**
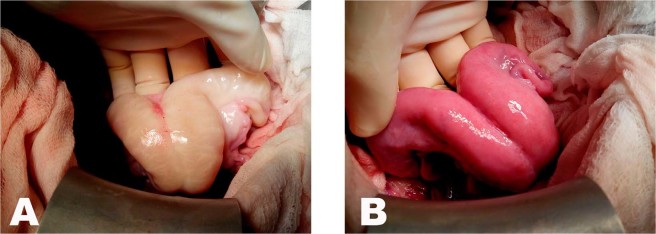
Photographs showing the colour of the ovine uterus before (**A**) and after (**B**) blood flow restoration.

Minimal bleeding occurred during the procedures, and the estimated loss of blood in all animals was 40 ml. All vaginal and vascular anastomosis were completed in all ten autotransplanted sheep. Recirculation of the grafts was satisfactory according to the pulsation observed through the anastomosed vessels and the colour change from pale to reddish (Fig. 5). This colour change occurred immediately, within 30 seconds to 1 min after the removal of the vascular clamps. The autotransplantation procedure was considered successful in all animals at the end of surgery.

### Surgical follow up: 15 days (T1), 1 month (T2) and 2 months (T3) post-transplantation

On day 3 after surgery the animals were in good health conditions. The median rectal temperature during the study was 39.1 ± 0.4 °C (no fever) and the same amount of food and water was consumed before and after transplantation.

In order to evaluate uterine structures, sequential transvaginal ultrasonography was performed at 2 weeks, 1 month and 2 months after surgery. In all transplanted ewes, the uterus with both horns and ovaries was identified, and the intensity of the myometrial and endometrial signals was normal, except for one animal at T2. Doppler Ultrasonography revealed no stasis in any of the vascular anastomoses, indicating that the graft had satisfactory recirculation, in nine out of ten ewes. The angiographies showed patency of all arterial anastomosis in all animals except for one at T2, which revealed a completely total lack of blood flow in the uterine arteries. In addition, one of the animals developed an aneurysm in the right uterine artery, which was observed for the first time at T2 angiography.

Vaginal examination with a hysteroscope showed a pinkish cervix and vagina and no lesion in the vaginal mucosa. As expected, it was impossible to assess the vaginal anastomosis site due to the morphologic characteristics of the ovine cervix, which does not allow a hysteroscope to pass through it. In three out of ten animals of the study, the uterine cervix was completely closed.

An exploratory laparoscopy was also performed one month after the transplant procedure. Eight animals encountered abdominal adhesions; however, in three of them the extent of adhesions was remarkable. In order to avoid damaging adjacent organs, these cases were approached with meticulous sharp dissections. The sites of vaginal and vascular anastomosis appeared to be healed completely with a filled utero-ovarian vein and a pulsating uterine artery. In addition, gross morphology, colour, texture and size of the grafts were normal in all animals except for one. The uterus of that ewe had a pale yellowish colouring, hard consistency and a fibrous appearance and belonged to the same animal that showed the abnormalities in the angiographic and ultrasound examinations. This individual was not excluded from the study but was euthanized for postmortem analysis. In the postmortem examination that followed, it was reported the arterial vasculature thrombi of this uterus.

### Fertility and offspring

The nine remaining transplanted ewes and the 5 ewes of the control group underwent artificial insemination after hormonal induction.

Examinations were carried out to determine pregnancies at day 40 after insemination. In all control animals and in six out of nine transplanted ewes, the abdominal ultrasound indicated pregnancies. The values of serial fetal biparietal diameter and renal length measurements were in accordance with the fetuses’ gestational age displaying no major differences between groups. Two ewes of the control group gave birth to healthful female lambs (on day 140 and 141 of pregnancy) before the planned date for caesarean section. On day 141 of pregnancy, one transplanted ewe showed signs of labour initiation with unrest behavior, regular abdominal contractions and vaginal dilation. After 8 hours, abdominal contractions stopped and a caesarean section was carried out. The fetus was dead and showed no signs of maceration or congenital malformations. The remaining pregnant animals (control and transplanted) had a cesarean section on the 142 day of gestation, all delivering a singleton pregnancy. The lambs were appropriately developed according to their estimated gestational age, sibling number (one in all cases) and sex, showing vital signs. Heart rate, fetal body weight and crown-rump length were found to be similar in the newborn lambs of the control and transplanted groups, with no significant differences.

### Macroscopic inspection and Histology

There was a good correlation between the laparoscopic images obtained at one-month follow-up and the macroscopic inspection findings in the transplanted animals, with the exception that in the gross inspection we found more abdominal adhesions. In the transplanted group, including the non-pregnant ewes, macroscopically, both uterine and ovarian morphology, colour and consistency were similar to those of the control animals, and patent vascular anastomoses were found. However statistically significant differences regarding abdominal adhesions were found between the two groups, being the abdominal adhesions more remarkable in the transplanted group. All the transplanted uteri revealed minor stenosis at the vaginal anastomosis, that did not show the control group.

**Figure 6:**
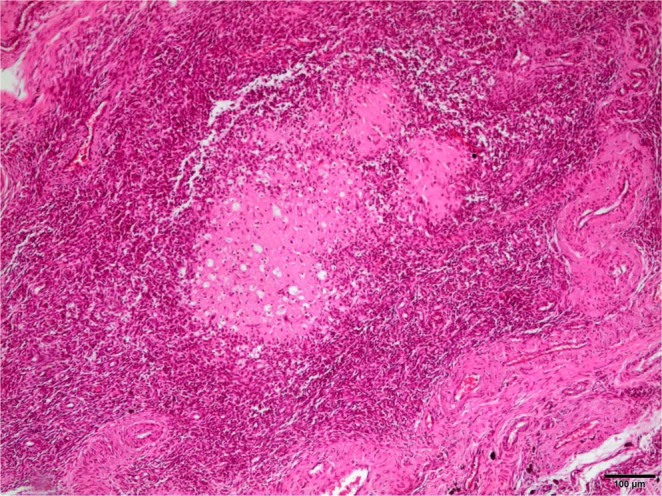
x10 Hematoxylin-eosin stain. Necrosis in an endometrial caruncle.

**Figure 7:**
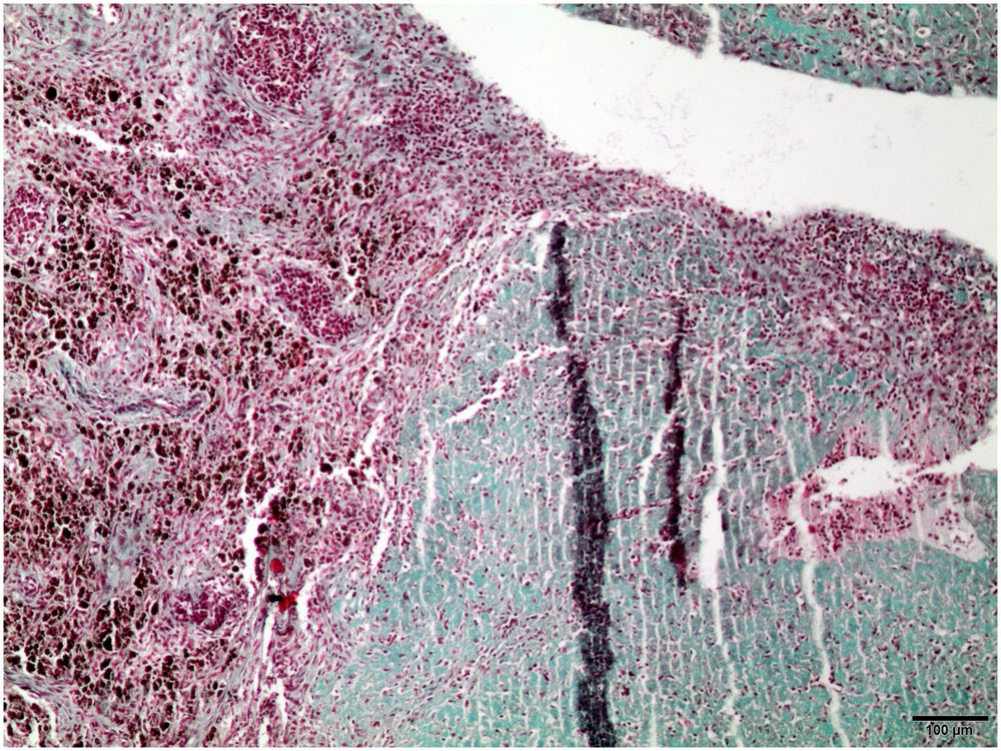
x10 Masson’s trichrome with light green stain. Macrophages full of hemosiderin in endometrium.

The histological report described a normal architecture of the uterus and cervix in the samples of both groups. No apoptosis in cell fragments, necrosis, vascular stasis, edema or arteritis were observed in any of the samples, except for one in which we observed some areas of necrosis in the endometrium (Fig. 6) and numerous macrophages full of hemosiderin (Fig. 7). This sample belonged to the pregnant transplanted animal that went into labour and finally suffered a miscarriage. Transplanted uteri revealed patchy areas of fibrosis along the uterine wall, unlike the control group.

## Discussion

Recent advances in assisted reproductive technology, such as *in vitro* fertilization (IVF), hormone stimulation, and intracytoplasmic sperm inoculation (ICSI) have improved infertility treatments in both males and females, but women with absolute uterine factor infertility (AUFI) still remain untreated. Women suffering from AUFI either have an absence of the uterus or a malformation (acquired congenitally or surgically) that interferes with the implantation of the embryo or the ability to carry a pregnancy to term^13,18^. An estimated 1 in 500 women of reproductive age experience AUFI^12^, which affects almost 1.5 million women of the world population^15,19^. The only way women with AUFI could have children until recently, was through adoption or gestational surrogacy^15^.

While many patients may be satisfied with these alternatives, adoption and gestational surrogacy might be inaccessible or unacceptable to others, due to restrictive adoption or surrogacy laws, cultural norms, personal values, or other ethical and financial reasons^20^. Thus, uterus transplantation has become a brand new potential treatment for these females with AUFI, and furthermore, the eleven live births after human uterus transplantation that have been reported^2–4^, are a proof of concept based on facts, for women with this kind of infertility.

After the first successful kidney transplantation^21^ more than half a century ago, transplantation surgery developed extensively. In addition, the introduction of cyclosporine in the 1980s resulted in significantly improved organ transplant survival^22^. Constant progress has been made in organ transplantation, microvascular anastomosis and immunosuppressive drugs over the last decade, making it possible for organ transplantation surgery to be no longer restricted to those who depend on these vital organs to stay alive. Transplantation surgery has also extended to include other organs or tissues which will improve patients quality of life^23^ such as face^24^, hand^25,26^, abdominal wall^27^, and larynx^28^ transplantation. Uterus transplantation is now included within this new quality-of-life group improving transplants^19,29,30^ as its main objective is to treat infertility, which is in turn linked to a deterioration of quality-of-life^31^. This case is also considered as a potentially life-giving transplantation. In addition, uterus transplantation is the first temporary organ transplantation, since the allograft would just be maintained during a given period of time, i.e., until the recipient had achieved the desired family size. So, this reduced graft time exposure would minimize the side effects of immunosuppressive medications in the long term.

Just like as other organs, transplantation of the uterus can be done with live or deceased donors, although so far the birth of a child has occurred only subsequent to living-donor transplants. A living donor is advantageous because it enables surgery to be scheduled according to optimal health conditions of both the ideal donor and the recipient. Furthermore, the operating rooms of donors and recipients can be nearby, which is relevant to substantially minimize cold ischemia time, thereby reducing the risk of graft complications after transplantation and increasing the graft survival rate. However, because a live donor’s uterine procurement involves anaesthetics risks and operational complications^5,6,20,32^, we believe that uterine recovery with a minimally invasive approach would be a more appropriate ethical and safe method.

Minimally invasive surgery is often used in various medical fields, including gynaecological and transplant surgery. Several authors have proved that minimally invasive surgical techniques used for the retrieval of grafts obtained from living donors realize similar results as open surgery, while providing the donor with the additional advantages^8^. In fact, laparoscopic surgery has been commonly used in the organ procurement procedure for liver, renal, and pancreas live-donor transplantations, resulting in a safe and useful method for the donor, with a reduced hospital stay and morbidity^8^. Almost all uterus transplantation studies conducted so far, either in animal models or humans, were carried out by open surgery except for a recent single case of uteri retrieval, using robotic or laparoscopic assistance^11,33^.

While research in the uterus transplantation topic has been carried out in several experimental models, including rodents^34–41^, large domestic species^29,42–56^, and lately nonhuman primates^57–61^, the first observations and key tests were carried out in rodents, and the findings of these trials were then applied in large domestic animal studies, with vasculature and dimensions of pelvic organs closer to human^16^.

The ovine model has been used by other authors in the field of uterus transplantation^42,44–54^. This model is one of the most commonly developed in translational gynaecological and paediatric research and surgical education, and It is becoming a progressively reliable alternative to models of canine and non-human primates and other smaller models, such as rodents. The ovine model is not only very suitable for its reproductive functional and physiological similarities with humans^62,63^, but also for its availability, reduced cost and ethical restrictions on other species. In our case and in addition to the above, we developed an ovine model for uterus transplantation since the foetal growth in this domestic animal is well-known. This fact would allow us to evaluate the foetal development during the gestational follow-up period. It is worth noting that all these previously mentioned characteristics to describe the sheep animal model can also be attributed to the porcine model, except for the anatomy of the reproductive system. The Swedish uterus transplantation research team used a porcine model in their first efforts to achieve a large animal uterine transplant model, and the success rate was below 20%^55^. This low rates of success in pig transplants were probably due to the very small caliber of the anastomosed uterine arteries and veins, and the higher thrombosis rate associated with these sites^46,64^.

An important issue in experimental transplantation research is to prevent as many harmful events as possible resulting in failed transplantation. These potential risky events mainly concern the surgeries at organ procurement and transplantation, ischaemia-reperfusion, rejection and effects of immunosuppressive drugs. Like other authors^46–49,51,53,54^, we performed an autotransplantation to prevent the potential detrimental effects of rejection and immunosuppression, but we also assessed the feasibility of laparoscopic organ retrieval the surgical technique. The present study is the first trial performed in animal, resulting in successful orthotopic transplantation of uterine vascular anastomosis after laparoscopic graft retrieval. Several animal studies have described orthotopic autotransplantation of the uterus by either vascular anastomosis^47–49,51,53,54,61,65^ or omentopexy^66–68^ with good results. We agree, though with Dahm-Kähler *et al*.^46^ and Wei *et al*.^52^ who questioned whether adequate uterine blood flow would be restored in an organ of this size before major necrosis occurred, using other techniques than vascular anastomosis. Thus, omental wrapping neovascularization does not provide the conditions necessary for the normal function of the transplanted uterus. This opinion is also supported by a dog study, that showed that the autotransplanted uterus could not be saved with neovascularization through omentopexy^69^. In our study we performed an end-to-end pattern to accomplish the vascular anastomosis of the uterine vessels^44,45,52,55,56^, but this method is limited to the small number of human cases where hysterectomy is performed simultaneously to the recipient^30^.

Another important issue in organ transplantation is the ischaemia-reperfusion damage. Transplanted organs can occur to be injured both during the ischaemic phase, when the organ is free of circulation, and when it is exposed to blood flow following prolonged ischaemia. Two warm ischaemic episodes (organ recovery and vascular anastomosis) and one cold ischaemic period (organ storage) are present. Due to the partial protection provided by the preservation solution remaining in the graft and the gradual increase in organ temperature^70^, the second warm ischaemic period is considered less harmful. Ischaemia-reperfusion injury affects transplantation success, which correlates with reduced post-transplant perfusion^71^, delayed graft function^72^, augmented rejection frequency^73^ and increased chronic rejection^74^. Therefore, any possible steps should be taken to reduce the severity of this injury. During organ storage, lower temperature is used to minimize ischaemic damage and therefore reperfusion injury. In addition, the blood of the donor is flushed out and it is restored by a protecting buffer to decrease the presence of an inflammatory reaction and to provide antioxidants, metabolic precursors and colloids^75^.

In our study, following the usual guidelines for organ transplantation, we maintained the uterus at 4 °C during the back-table surgery. A number of preservation solutions are commercially available and are all designed to reduce loss of cellular homeostasis, to provide antioxidant reperfusion defence and allow for rapid rescue from ischaemic energy stores depletion^75^. In the uterus context of transplantation, many preservation solutions such as Wisconsin^42,45^, Celsior^49,50^, Perfadex^47,48^, Histidine-tryptophan-ketoglutarate (HTK)^44,51,52^, Ringer’s acetate^46,47^, heparinized Ringer’s lactate^53^, and heparinized saline^54^ have been described to be used. We used heparinized saline solution to flush the uterus and preserve it during the back-table surgery and our mean duration of the cold ischaemia and warm ischaemia were similar to those described by other authors^50,51^ who performed their studies in an ovine model too. As the Swedish team did^46–48,50,51^ we sutured the vaginal anastomosis after removing the vascular clamps in order to reduce the duration of the second warm period. Although the main processes of cellular response to hypothermia, ischemia and reperfusion are similar for all cell types^76,77^, there is a large variety of ischaemic tolerability among different organs. For instance, it has been shown in clinical practice that the kidney can endure cold ischaemia time up to 36 hours, whereas the heart is under 6 hours^30^. This difference in ischaemic tolerance and reperfusion can be attributed to a variation of organ characteristics, such as energy requirement, parenchymal cell activity and resident populations of immune-cells^47^. Recommended ischaemia times in uterus transplantation are not known yet, however, studies of uterine transplantation ischaemia-reperfusion damage show that this organ is quite resistant to ischaemia^37,47,78^.

Regarding the graft procurement total surgical time, our results showed lower values compared to those reported in a series of conventional open surgery graft retrieval in ovine model^47,51,52^. Moreover, our execution times were shorter even though our graft procurement total surgical time included specific laparoscopic surgery manoeuvres such as pneumoperitoneum creation and trocar insertion. This could indicate that laparoscopic removal of the organ in uterus transplantation reduces the total time invested in surgical graft procurement, just as Johannensson *et al*.^1^ suggested. However, when we contrasted our results with past reports of uterus transplantation in ewes using an aortocava patch^50^, our times were higher. This may have been due to the fact that these authors harvested the whole internal reproductive organs ‘en bloc’ (involving the abdominal aortocava vessels, the internal iliac artery and vein, both uterine and ovarian arteries as well as utero-ovarian veins) and to the use of an endostapler to transect the sigmoid colon in order to save time for the exposure and removal of the entire intact vascular bifurcation. On the other hand, mean total surgery time (retrieval plus transplant) was lower in our study when compared with the mean total surgery times reported by Wranning *et al*.^47,48^, Wei *et al*.^52^, Saso *et al*.^51^ who performed the whole surgery with the open technique. In contrast, comparing our results with those reported by Ramirez *et al*.^44^ and Gauthier *et al*.^50^ who also performed the whole surgery with the open technique, our mean total surgery time is higher. This is basically because they carried out allotransplantations instead of autotransplantations. In general, performing an autotransplant takes longer time of surgery, mainly because both organ recovery and organ transplantation are performed by the same surgeon^16^.

During the surgery and the immediate postoperative period, there were no complications, a fact that highlights the surgical safety of combining the microsurgical transplantation with the laparoscopic approach.

We used angiography, transvaginal ultrasound, abdominal colour Doppler ultrasound, vaginoscopy, and exploratory laparoscopy to assess the vascular patency and graft viability. Other authors have used the ultrasound^52^, the vaginoscopy^48,50^, and the exploratory laparoscopy^52^ for graft assessment; however, we are the first ones to introduce the angiography for that purpose. On the other hand, magnetic resonance imaging^50^ and indocyanine green fluorescence imaging^60,79,80^ have been also described to evaluate the transplanted uteri.

It is essential to mention that the expression ‘successful uterus transplantation’ should include restarted uterine cycle and also the complete capability for terminating the pregnancy and giving birth to live offsprings^16^. For this reason, one of the aims of our study is to determine fertility after uterus transplantation. There are only two previous reports of graft fertility after transplantation in the ovine model^44,48^. One study tested the fertility of the transplanted uteri after natural mating and pregnancy occurred in 60% of the transplanted mated ewes. These pregnancies ended up in one miscarriage and two healthy lambs^48^. In the second study, five transplanted ewes were subjected to embryo transfer. Among these, three pregnancies occurred with the outcome of one ectopic pregnancy, one miscarriage and one live birth^44^. Our fertility results were similar, but slightly better than the previously described. In our study, six of the transplanted ewes became pregnant after artificial insemination and resulted in one miscarriage and five live births. The lambs were similar in size to those of the ewes control group.

Regarding the histopathological findings, in all the samples there was an absence of necrosis, oedema, apoptotic cell fragments, vascular stasis and arteritis, which are all acute markers of cellular damage, we would like to indicate that there was no acute ischaemic and reperfusion damage in the uterus when the samples were taken. Nevertheless, the transplanted uteri showed patchy areas of fibrosis along the uterine wall when compared to the controls. This could be explained by the fact that there was an ischaemic and reperfusion injury after the transplantation surgery, as fibrosis is a chronic marker of tissue damage.

## Conclusions

The present study highlights the promising application of laparoscopy for graft procurement surgery in uterine transplantation from living donors for absolute uterine infertility treatment. Laparoscopy seems to be a safe, time saving, useful and technically feasible surgical procedure for uterus extraction, with the additional benefits of minimally invasive surgery. It was also demonstrated that after a successful uterine transplant, pregnancy can be achieved by combining laparoscopy for uterine retrieval and microsurgery for vascular anastomosis. With the increasing success rate of human uterus transplantation trials from living donors, we believe that laparoscopic graft extraction will soon become a reality in the transplantation field. However, uterus transplantation will remain as a clinical experimental procedure until sufficient experience is achieved.

## Data Availability

On reasonable request, data sets generated during the current study -or those analyzed during it- are available from the corresponding author.

## References

[1] Johannesson L, Jarvholm S (2016). Uterus transplantation: current progress and future prospects. International journal of women’s health.

[2] Brannstrom, M. *et al*. Uterus transplantation: A Rapidly Expanding Field. *Transplantation* (2017). 10.1097/TP.0000000000002035 29210893

[3] Testa G (2018). First live birth after uterus transplantation in the United States. Am J Transplant.

[4] Soares, J. M. J., Ejzenberg, D., Andraus, W., D’Albuquerque, L. A. & Baracat, E. C. First Latin uterine transplantation: we can do it! *Clinics* **71**, 627–628, (11)01 (2016). 10.6061/clinics/2016(11)01 PMC5108168 27982161

[5] Brannstrom M (2014). First clinical uterus transplantation trial: a six-month report. Fertil Steril.

[6] Lavoue V (2017). Which Donor for Uterus Transplants: Brain-Dead Donor or Living Donor? A Systematic Review. Transplantation.

[7] Brannstrom M, Dahm-Kahler P, Kvarnstrom N (2018). Robotic-assisted surgery in live-donor uterus transplantation. Fertil Steril.

[8] Berloco PB (2011). Laparoscopy in solid organ transplantation: a comprehensive review of the literature. Il Giornale di chirurgia.

[9] Wang Y, Deng L, Cao L, Xu H, Liang Z (2015). The Outcome of Laparoscopy Versus Laparotomy for the Management of Early Stage Cervical Cancer-Meta Analysis. Journal of minimally invasive gynecology.

[10] Doumerc N, Beauval JB, Rostaing L, Sallusto F (2016). A new surgical area opened in renal transplantation: a pure robot-assisted approach for both living donor nephrectomy and kidney transplantation using transvaginal route. Transpl Int.

[11] Wei L (2017). Modified human uterus transplantation using ovarian veins for venous drainage: the first report of surgically successful robotic-assisted uterus procurement and follow-up for 12 months. Fertil Steril.

[12] Milliez J (2009). Uterine transplantation FIGO Committee for the Ethical Aspects of Human Reproduction and Women’s Health. International journal of gynaecology and obstetrics: the official organ of the International Federation of Gynaecology and Obstetrics.

[13] Hanafy A, Diaz-Garcia C, Olausson M, Brannstrom M (2011). Uterine transplantation: one human case followed by a decade of experimental research in animal models. The Australian & New Zealand journal of obstetrics & gynaecology.

[14] Brannstrom M, Diaz-Garcia C, Johannesson L, Dahm-Kahler P, Bokstrom H (2015). Livebirth after uterus transplantation - Authors’ reply. Lancet.

[15] Dahm-Kahler P, Diaz-Garcia C, Brannstrom M (2016). Human uterus transplantation in focus. British medical bulletin.

[16] Brannstrom M, Diaz-Garcia C, Hanafy A, Olausson M, Tzakis A (2012). Uterus transplantation: animal research and human possibilities. Fertil Steril.

[17] Council, N. R. Guide for care and use of laboratory animals. The National Academies Press, Washington, DC (2010).

[18] Brannstrom M (2017). Uterus transplantation and beyond. Journal of materials science. Materials in medicine.

[19] Brannstrom M (2015). Uterus transplantation. Current opinion in organ transplantation.

[20] Flyckt R (2018). Uterine Transplantation: Surgical Innovation in the Treatment of Uterine Factor Infertility. Journal of obstetrics and gynaecology Canada: JOGC = Journal d’obstetrique et gynecologie du Canada: JOGC.

[21] Merrill JP, Murray JE, Harrison JH, Guild WR (1956). Successful homotransplantation of the human kidney between identical twins. Journal of the American Medical Association.

[22] Cyclosporin in cadaveric renal transplantation: one-year follow-up of a multicentre trial (1983). Lancet.

[23] Siemionow MZ, Kulahci Y, Bozkurt M (2009). Composite tissue allotransplantation. Plastic and reconstructive surgery.

[24] Devauchelle B (2006). First human face allograft: early report. Lancet.

[25] Dubernard, J. M. *et al*. The first transplantation of a hand in humans. Early results. *Chirurgie; memoires de l’Academie de chirurgie* **124**, 358–365; discussion, 365–357 (1999). 10.1016/s0001-4001(00)80007-0 10546388

[26] Dubernard JM (1999). Human hand allograft: report on first 6 months. Lancet.

[27] Levi DM (2003). Transplantation of the abdominal wall. Lancet.

[28] Birchall MA (2006). Laryngeal transplantation in 2005: a review. Am J Transplant.

[29] Sieunarine K (2005). Possibilities for fertility restoration: a new surgical technique. International surgery.

[30] Brannstrom M, Wranning CA, Altchek A (2010). Experimental uterus transplantation. Human reproduction update.

[31] Chachamovich JR (2010). Investigating quality of life and health-related quality of life in infertility: a systematic review. Journal of psychosomatic obstetrics and gynaecology.

[32] Fageeh W, Raffa H, Jabbad H, Marzouki A (2002). Transplantation of the human uterus. International journal of gynaecology and obstetrics: the official organ of the International Federation of Gynaecology and Obstetrics.

[33] Puntambekar S (2018). Laparoscopic-Assisted Uterus Retrieval From Live Organ Donors for Uterine Transplant. J Minim Invasive Gynecol.

[34] Wranning CA (2007). Rejection of the transplanted uterus is suppressed by cyclosporine A in a semi-allogeneic mouse model. Human reproduction.

[35] El-Akouri RR, Molne J, Groth K, Kurlberg G, Brannstrom M (2006). Rejection patterns in allogeneic uterus transplantation in the mouse. Human reproduction.

[36] Racho El-Akouri R (2002). Heterotopic uterine transplantation by vascular anastomosis in the mouse. The Journal of endocrinology.

[37] Racho El-Akouri R, Wranning CA, Molne J, Kurlberg G, Brannstrom M (2003). Pregnancy in transplanted mouse uterus after long-term cold ischaemic preservation. Human reproduction.

[38] Racho El-Akouri R, Kurlberg G, Brannstrom M (2003). Successful uterine transplantation in the mouse: pregnancy and post-natal development of offspring. Human reproduction.

[39] Diaz-Garcia C, Akhi SN, Wallin A, Pellicer A, Brannstrom M (2010). First report on fertility after allogeneic uterus transplantation. Acta obstetricia et gynecologica Scandinavica.

[40] Wranning CA, Akhi SN, Diaz-Garcia C, Brannstrom M (2011). Pregnancy after syngeneic uterus transplantation and spontaneous mating in the rat. Human reproduction.

[41] Diaz-Garcia C, Akhi SN, Martinez-Varea A, Brannstrom M (2013). The effect of warm ischemia at uterus transplantation in a rat model. Acta obstetricia et gynecologica Scandinavica.

[42] Gonzalez-Pinto IM (2013). Uterus transplantation model in sheep with heterotopic whole graft and aorta and cava anastomoses. Transplantation proceedings.

[43] Avison DL (2009). Heterotopic uterus transplantation in a swine model. Transplantation.

[44] Ramirez ER (2011). Pregnancy and outcome of uterine allotransplantation and assisted reproduction in sheep. Journal of minimally invasive gynecology.

[45] Ramirez ER, Ramirez DK, Pillari VT, Vasquez H, Ramirez HA (2008). Modified uterine transplant procedure in the sheep model. Journal of minimally invasive gynecology.

[46] Dahm-Kahler P (2008). Transplantation of the uterus in sheep: methodology and early reperfusion events. The journal of obstetrics and gynaecology research.

[47] Wranning CA (2008). Transplantation of the uterus in the sheep: oxidative stress and reperfusion injury after short-time cold storage. Fertil Steril.

[48] Wranning CA (2010). Fertility after autologous ovine uterine-tubal-ovarian transplantation by vascular anastomosis to the external iliac vessels. Human reproduction.

[49] Tricard J (2017). Uterus tolerance to extended cold ischemic storage after auto-transplantation in ewes. European journal of obstetrics, gynecology, and reproductive biology.

[50] Gauthier T (2011). Uterine allotransplantation in ewes using an aortocava patch. Human reproduction.

[51] Saso S (2015). Achieving uterine auto-transplantation in a sheep model using iliac vessel anastomosis: a short-term viability study. Acta obstetricia et gynecologica Scandinavica.

[52] Wei L (2013). Modified uterine allotransplantation and immunosuppression procedure in the sheep model. PloS one.

[53] Andraus W (2017). Sheep Model for Uterine Transplantation: The Best Option Before Starting a Human Program. Clinics.

[54] Solomonov, E., Marcus Braun, N., Siman-Tov, Y. & Ben-Shachar, I. Team preparation for human uterus transplantation: Autologous transplantation in sheep model. *Clinical transplantation* **31** (2017). 10.1111/ctr.13137 29032587

[55] Wranning CA (2006). Auto-transplantation of the uterus in the domestic pig (Sus scrofa): Surgical technique and early reperfusion events. The journal of obstetrics and gynaecology research.

[56] Sieunarine K (2005). Is it feasible to use a large vessel patch with a uterine allograft en bloc for uterine transplantation?. International surgery.

[57] Adachi M (2016). Evaluation of allowable time and histopathological changes in warm ischemia of the uterus in cynomolgus monkey as a model for uterus transplantation. Acta obstetricia et gynecologica Scandinavica.

[58] Kisu I (2012). A new surgical technique of uterine auto-transplantation in cynomolgus monkey: preliminary report about two cases. Archives of gynecology and obstetrics.

[59] Mihara M (2012). Uterine autotransplantation in cynomolgus macaques: the first case of pregnancy and delivery. Human reproduction.

[60] Mihara M (2011). Uterus autotransplantation in cynomolgus macaques: intraoperative evaluation of uterine blood flow using indocyanine green. Human reproduction.

[61] Enskog A (2010). Uterus transplantation in the baboon: methodology and long-term function after auto-transplantation. Human reproduction.

[62] Dobrowolski W, Hafez ES (1970). Ovariouterine vasculature in sheep. American journal of veterinary research.

[63] Janson PO (1983). Blood flow in the ovary and adjacent structures of the non-pregnant sheep. Acta endocrinologica.

[64] Brannstrom M (2015). The Swedish uterus transplantation project: the story behind the Swedish uterus transplantation project. Acta obstetricia et gynecologica Scandinavica.

[65] Johannesson L (2012). Uterus transplantation in a non-human primate: long-term follow-up after autologous transplantation. Human reproduction.

[66] Zhordania IF, Gotsiridze OA (1964). Vital Activity of the Excised Uterus and Its Appendages after Their Autotransplantation into Omentum. Experimental Research. Acta chirurgiae plasticae.

[67] O’Leary JA, Feldman M, Gaensslen DM (1969). Uterine and tubal transplantation. Fertil Steril.

[68] Scott JR, Pitkin RM, Yannone ME (1971). Transplantation of the primate uterus. Surgery, gynecology & obstetrics.

[69] Paldi E, Gal D, Barzilai A, Hampel N, Malberger E (1975). Genital organs. Auto and homotransplantation in forty dogs. International journal of fertility.

[70] Feuillu B (2003). Kidney warming during transplantation. Transpl Int.

[71] Menger MD, Rucker M, Vollmar B (1997). Capillary dysfunction in striated muscle ischemia/reperfusion: on the mechanisms of capillary “no-reflow”. Shock.

[72] Quiroga I (2006). Major effects of delayed graft function and cold ischaemia time on renal allograft survival. Nephrology, dialysis, transplantation: official publication of the European Dialysis and Transplant Association - European Renal Association.

[73] Totsuka E (2004). Synergistic effect of cold and warm ischemia time on postoperative graft function and outcome in human liver transplantation. Transplantation proceedings.

[74] Schwarz A (2005). Risk factors for chronic allograft nephropathy after renal transplantation: a protocol biopsy study. Kidney international.

[75] Muhlbacher F, Langer F, Mittermayer C (1999). Preservation solutions for transplantation. Transplantation proceedings.

[76] Boutilier RG (2001). Mechanisms of cell survival in hypoxia and hypothermia. The Journal of experimental biology.

[77] Jassem W, Fuggle SV, Rela M, Koo DD, Heaton ND (2002). The role of mitochondria in ischemia/reperfusion injury. Transplantation.

[78] Wranning CA, Molne J, El-Akouri RR, Kurlberg G, Brannstrom M (2005). Short-term ischaemic storage of human uterine myometrium–basic studies towards uterine transplantation. Human reproduction.

[79] Kisu I (2013). Indocyanine green fluorescence imaging in the pregnant cynomolgus macaque: childbearing is supported by a unilateral uterine artery and vein alone?. Archives of gynecology and obstetrics.

[80] Kisu I (2012). Indocyanine green fluorescence imaging for evaluation of uterine blood flow in cynomolgus macaque. PloS one.

