# Author Correction: Laparoscopic uterine graft procurement and surgical autotransplantation in ovine model

**DOI:** 10.1038/s41598-021-90396-z

**Published:** 2021-05-25

**Authors:** Francisco Miguel Sánchez-Margallo, Belén Moreno-Naranjo, María del Mar Pérez-López, Elena Abellán, José Antonio Domínguez-Arroyo, José Mijares, Ignacio Santiago Álvarez

**Affiliations:** 1 Laparoscopy Department, Jesús Usón Minimally Invasive Surgery Centre, 10071 Cáceres, Spain; 2 Microsurgery Department, Jesús Usón Minimally Invasive Surgery Centre, 10071 Cáceres, Spain; 3 Instituto Extremeño de Reproducción Asistida (IERA), 06006 Badajoz, Spain; 4 Assisted Reproduction Unit, Jesús Usón Minimally Invasive Surgery Centre, 10071 Cáceres, Spain; 5 Anatomy and Cell Biology Department, School of Medicine, University of Extremadura, 06071 Badajoz, Spain

Correction to: *Scientific Reports* https://doi.org/10.1038/s41598-019-44528-1, published online 30 May 2019

In the original version of this Article, the author Ignacio Santiago Álvarez was incorrectly indexed.

The original Article has been corrected.

